# Editorial: Therapeutic process and treatment evaluation in forensic psychiatry and prison

**DOI:** 10.3389/fpsyt.2022.1095592

**Published:** 2022-11-25

**Authors:** Manuela Dudeck, Jürgen Leo Müller, Birgit Völlm, Najat R. Khalifa, Judith Streb

**Affiliations:** ^1^Department of Forensic Psychiatry and Psychotherapy, Ulm University, Ulm, Germany; ^2^Clinic for Psychiatry and Psychotherapy – Forensic Psychiatry, Human Medical Center Göttingen, Georg-August-University Göttingen, Göttingen, Germany; ^3^Department of Forensic Psychiatry, University Medicine Rostock, Rostock, Germany; ^4^Department of Psychiatry, Queen's University, Kingston, ON, Canada

**Keywords:** forensic psychiatry, prison, risk assessment, recidivism, treatment effectiveness, offender treatment programmes

In forensic psychiatry and correctional settings, treatment approaches have two broad goals: to prevent crime and, when applicable, to treat an underlying mental illness. In the context of crime prevention, forensic rehabilitation models, such as the Risk-Need-Responsivity (RNR) Model (1), have been devised to reduce re-offending rates. In programmes based on RNR risk factors are identified and coping strategies are practiced. Studies show that programmes operating according to these principles lead to a significant reduction in recidivism (2). In addition, standardized professional risk assessment tools have been developed and their use has become increasingly important. These instruments can be used to inform decisions about different situations. For example, the decision whether someone should be placed in a forensic psychiatry hospital or prison setting is based on the general likelihood of recidivism, so the probability of an adverse incident occurring during detention needs to be predicted. On the other hand, to justify discharge from a forensic psychiatric institution or release from prison according to § 66 of the German Criminal Code, the necessary conditions for preventing recidivism have to be anticipated. The questions to be answered in these assessments not only have a different focus but also apply to different time periods, and different predictors are of relevance depending on the time of the assessment. The assessment of long-term risk considers actuarial and dynamic risk variables [e.g., Historical Clinical Risk Management-20, Version 3, HCR-20 v3; (3)]. In contrast, the assessment of short-term risk focuses on variables that can become a significant indicator of risk for the person being assessed if they occur repeatedly, in the sense of a crisis-like escalation; the importance of such variables should not be overlooked [e.g., Short-Term Assessment of Risk and Treatability, START; (4)]. Although a number of well-studied instruments are available for prognosis after release or discharge, there is a relative dearth of empirical data on the quality of predictors for short-term prognosis and continuous risk assessments. The START method (4) captures dynamic variables that are scored as either risk factors or protective factors, depending on whether they are present and their degree of expression. START is a clinical guide for risk domains related to negative behaviors, such as violence to others, suicide, self-harm, self-neglect, unauthorized absence (e.g., failure to return from a day pass), substance use, risk of being victimized and general offending.

In this volume, two papers present studies that tested the applicability of START. Driven by the awareness that the risk of violence and other undesirable behaviors are of a major concern in forensic psychiatric facilities, Hvidhjelm et al. studied the utility of START in preventing these critical events in forensic units in Denmark. They studied time periods in which they used START in patients and compared it to control periods in which START was not used. Comparing the rate of mechanical restraints within and outside START periods, they found that the rate of mechanical restraint use within the START period was significantly lower (82%). Hvidhjelm et al. identified benefits and outcomes of the implementation of START, particularly in relation to the use of mechanical restraint in a forensic setting. With regard to cultural differences in the predictive accuracy of assessment tools, Kikuchi et al. investigated the benefit of the START in the Japanese Forensic Probation Service. They found that START was able to predict physical violence and unauthorized leave as well as self-neglect. Their results allow to recommend the START for treatment planning and promotion of recovery in the Japanese Forensic Probation Service.

Severe mental disorders are highly prevalent among patients in forensic psychiatric and prison settings. The approximate prevalence rates of severe mental disorders amongst prison inmates are as follows: psychotic illnesses, 4%; major depression, 10%; and personality disorders, 50% (5), alcohol use disorder, 24%; drug use disorder, 30% (6). It is imperative that these individuals are offered evidence-based therapies, and extensive guidelines from the respective professional societies are available for the most common disorders. There is a broad consensus among researchers and clinicians that patient and treatment programme characteristics should be matched to optimize treatment outcomes. However, no consensus exists on the question of which specific factors should be considered. The following studies focus on internal (e.g., characteristics of the patient) and external factors (e.g., characteristics of the therapy and treatment setting) that may hinder or enhance the therapeutic process.

So far, research on psychopharmacological treatments for forensic patients with schizophrenia has mainly focused on men. However, many countries have seen an increase in the number of women hospitalized in forensic psychiatry settings, underlining the need for evidence-based research on sex-specific treatment strategies for female forensic patients (7). Mayer et al. surveyed psychopharmacological treatment strategies, psychopathological characteristics and neurological and metabolic adverse effects of treatment in 29 male and 29 female forensic-psychiatric patients. They found that, compared to men, women had more severe mental disorders and were more frequently treated with second-generation depot antipsychotics. However, the researchers found no differences between the sexes in the efficacy of the dosages.

Although opioid agonist treatment (OAT) is the first line recommended treatment for opioid use disorders in the relevant guidelines, in contravention of the principle of equivalence, this treatment is often not available to prisoners or patients in forensic-psychiatric care. Reiners et al. surveyed all forensic-psychiatric hospitals offering treatment for patients with substance use disorders in Germany and found that only under half offered such interventions. Critical incidents, such as violence or absconding, did not differ between clinics that did and did not offer OAT. Maybe somewhat surprising, early termination of treatment (or treatment dropout) was higher in clinics with OAT. A high proportion of terminations were due to rule violations such as giving the OAT away. Other reasons included additional drug use and refusal to give a urine drug sample. It is possible that those who received OAT represented a patient group with more complex needs and hence achieved less favorable outcomes. More research is clearly needed in order to understand OAT practice and risks.

Conducting studies on the efficacy of specific therapeutic approaches to reduce recidivism rates is challenging, not least because of the difficulties inherent in empirically demonstrating the superiority of any particular treatment approach over usual care. Lardén et al. used a randomized controlled design to evaluate the effectiveness of an individual Cognitive-Behavioral Intervention (iCBT) for serious young male violent offenders in comparison to treatment-as-usual (TAU). After 24 months, the violent reconviction rate was slightly higher for iCBT+TAU vs. TAU-only group. The authors emphasized that these differences were not significant, nevertheless they did not find an additive effect of individual CBT beyond group-based TAU. They discussed the impact of sample size and substantial treatment dropouts on outcomes.

Sociotherapeutic treatment comprises psychotherapeutic, educational, vocational and recreational measures in the context of a milieu-therapeutic setting. In Germany, sociotherapeutic treatment is offered in special facilities within the prison system. Hausam et al. evaluated post-release recidivism in a group of male young offenders aged 14–22 years, having undergone treatment in a social-therapeutic unit to a group matched for offending not having been through this treatment. They found no main effect on recidivism. Additional analyses showed a significant effect of vocational training and education, but not individual psychology sessions on reoffending. These results have important implication for designing treatment programmes for juveniles.

About a quarter of all prison inmates have attention deficit hyperactivity disorder (ADHD). To better support these patients in the prison system, Buadze et al. surveyed 19 staff members of a correctional facility in Switzerland and evaluated their responses by content analysis. The results suggest that inmates with ADHD are perceived as being difficult and are also more likely to be subjected to disciplinary sanctions. The authors recommend providing training to staff so that ADHD can be diagnosed early and treated adequately (including by therapy and with drugs).

Psychological distress is common among prison inmates. The study by Sfendla et al. examined whether inmates' psychological distress was reduced when they participated in a weekly 90-min yoga class. A control group participated in free-choice physical exercise at the same time. Before and after the 10-week intervention, participants completed the Brief Symptom Inventory. Results showed that physical activity (including yoga) reduced levels of psychological distress but that the positive effect of yoga was even stronger than that of free-choice physical exercise with respect to symptoms of compulsion, paranoid ideation and somatization.

The physical environment has been described as one of the central determinants of mental health and wellbeing (8) and researchers of different disciplines have stressed the importance of a comprehensive understanding of the concepts of space and place for mental health and care (9). Ross et al. reviewed the literature on the relationship between the physical environment and wellbeing in prisons and secure forensic mental health settings. In addition, they report on theoretical models and findings from non-forensic mental health settings. Their findings highlight the link between overcrowding and aggression, as well as other measures of mental health and wellbeing. They also highlight the impact of architecture and designs of these institutions on these measures. The findings of this study signify the importance of achieving the right balance between security, therapy and rehabilitation in custodial and secure hospital settings.

To reduce recidivism, close networking and cooperation is necessary between patients/prison inmates, their families, facility staff, the courts and services providing aftercare to forensic psychiatric patients. This approach requires transparency and a good exchange of information between stakeholders. The last two studies in this volume focus on the care structure for mentally ill people. Askola et al. analyzed the need and development possibilities of forensic psychiatry in Finland. For this purpose, they interviewed forensic psychiatric patients and their parents, as well as service providers, and evaluated the responses by content analysis. Respondents called for increased risk awareness and risk assessment skills at the general psychiatric level, increased therapeutic engagement throughout the rehabilitative process and structured post-discharge aftercare. In 2019, the first psychiatric day hospital (PDC) was established in Switzerland to improve mental health care for pretrial detainees. Using a cross-sectional observational study design, Gerth et al. aimed to evaluate the need for mental health care in pretrial detention and the potential of the PDC in order to improve it. The findings revealed a significant reduction in psychiatric hospital admission rates (18.5 %) for pretrial detainees who were treated in the PDC. This group of detainees significantly differed from other prisoners in relation to mental disorder, gender and alleged index offense. More specifically, they were more likely than other groups to have adjustments disorders and less likely to have schizophrenia spectrum disorders. Collectively, the findings signify the role of innovative intervention like PDC in improving mental health outcomes for pretrial detainees.

## Author contributions

MD, NK, JM, BV, and JS wrote parts of the manuscript. JS combined the contributions and formulated the transitions. All authors read and approved the submitted version.

## Conflict of interest

The authors declare that the research was conducted in the absence of any commercial or financial relationships that could be construed as a potential conflict of interest.

## Publisher's note

All claims expressed in this article are solely those of the authors and do not necessarily represent those of their affiliated organizations, or those of the publisher, the editors and the reviewers. Any product that may be evaluated in this article, or claim that may be made by its manufacturer, is not guaranteed or endorsed by the publisher.
